# Editorial: the airborne microbiome - implications for aerosol transmission and infection control – special issue

**DOI:** 10.1186/s12879-019-4399-z

**Published:** 2019-08-29

**Authors:** Julian W. Tang, Yuguo Li

**Affiliations:** 1 Clinical Microbiology, University Hospitals of Leicester NHS Trust, Infirmary Square, Leicester, LE1 5WW UK; 2 Department of Mechanical Engineering, The University of Hong Kong, Haking Wong Building 7/F, Pokfulam Road, Pokfulam, Hong Kong SAR China

**Keywords:** Airborne, Aerosol, Transmission, Detection, Exhalation, Breath, Infection, Control

## Abstract

Although the title of the Special Issue is ‘Airborne Microbiome’ the manuscripts received have highlighted a variety of peripheral, yet related aspects of this. The contributions are a mixture of primary research, reviews and commentaries, including: new methods to explore environmental niches where such microbes may grow, their detection and characterisation in the human host, which pathogens are present in the respiratory tract and can be exhaled in human breath to potentially spread via the airborne route, and some strategies for their control. Finally, a historical-to-current overview explores human-microbial interactions, including problems with sampling and detection methods, drug resistance, the role of super-spreaders and issues around research funding.

Each time a journal puts out a call for a ‘Special Issue’ on one theme or another, it is never certain what type of response will be received. There may be little or no response at all, or an over-whelming response from which only a few articles can be selected, or, as in this case, there could be an enthusiastic response with a variety of articles from authors who have interpreted the ‘Special Issue’ theme in different ways.

Although the title of the Special Issue is ‘Airborne Microbiome’ the manuscripts received have highlighted a variety of peripheral, yet related aspects of this. The contributions are a mixture of primary research, reviews and commentaries, including: new methods to explore environmental niches where such microbes may grow [1], their detection and characterisation in the human host [2–4], which pathogens are present in the respiratory tract and can be exhaled in human breath to potentially spread via the airborne route [5, 6], and some strategies for their control [7]. Finally, a historical-to-current overview explores human-microbial interactions, including problems with sampling and detection methods, drug resistance, the role of super-spreaders and issues around research funding [8].

However it is specifically defined, the general concepts of the airborne microbiome, and the potential risks it can pose to human health, are now well-established amongst academics and the general public. The greatest fear when encountering any new or re-emerging pathogen is how and when it can be transmitted via the airborne route – yet the precise evidence to support such conclusions can be difficult to obtain and interpret in a definitive manner [9–12]. Such evidence and its interpretations are important as they are required to effectively inform infection control guidance, the most obvious being: what protective equipment is required when managing patients infected with pathogens that are potentially airborne-transmissible? [13–15]

Hence, it is in all our interests to continue performing studies to elucidate more precisely the various routes of transmission for different pathogens so that appropriate interventions can be applied when caring for those infected, to prevent secondary spread of these agents. Therefore, we hope that this Special Issue has raised more questions than answers and will inspire more researchers to explore the airborne microbiome in more detail, both for healthcare-related applications, but also, in a much wider sense, to better understand the world in which we all live – and the organisms with which we share it.

## Data Availability

Not applicable
